# First-pass success in video laryngoscopy with transcutaneous infrared illumination in patients with normal airways–a clinical pilot study

**DOI:** 10.1007/s10877-025-01361-4

**Published:** 2025-09-26

**Authors:** Abdulrahman Dardeer, Muhammad Firas Alhammad, Khaled J. Zaza, Anas N. Shallik, Yasser Ali Hammad, El-Sayed Mohamed Elkarta, Nabil A. Shallik

**Affiliations:** 1 Watford General Hospital, West Hertfordshire Teaching Hospitals NHS Trust, Watford, UK; 2 Aaesthesia, ICU and Perioperative Medicine Department, Hamad Medical Corporation, Doha, Qatar; 3 Royal College of Surgeons in Ireland Medical University of Bahrain, Manamah, Bahrain; 4 Clinical Anaesthesiology Department, Weill Cornell Medical College in Qatar, Doha, Qatar; 5 Clinical Anaesthesiology Department, College of Medicine, Qatar University, Doha, Qatar; 6 Damietta Faculty of Medicine, Al-Azhar University, Damietta, Egypt

**Keywords:** Transcutaneous infra-red illumination, Video laryngoscopy, First-pass succes, Infrared red retrograde intubation system, IRRIS

## Abstract

**Purpose:**

Endotracheal intubation is a critical skill in anesthesia, particularly for patients with compromised airways. This randomized pilot study evaluated the feasibility and impact of the Infrared Red Intubation System (IRRIS^®^) on video laryngoscopy performance, first-attempt success rate, and intubation time.

**Methods:**

Thirty patients were randomized into two groups: one with the IRRIS device and one without (control). The primary outcome was the impact of IRRIS on first-pass success. Secondary outcomes included glottic visibility, intubation time, and adverse effects.

**Results:**

Results showed that both groups demonstrated nearly identical percentages of glottic opening (POGO) and glottic entrance visibility, achieving successful intubation on the first attempt. Although the IRRIS group had a slightly longer intubation time and more instances of required external manipulation, the vocal cords were not visible without IRRIS in the most obese patient in our cohort.

**Conclusion:**

The IRRIS device effectively illuminated the laryngeal inlet, enhancing differentiation from surrounding structures, such as the esophagus. This study suggests that IRRIS may be a valuable adjunct for video laryngoscopy in patients with difficult airways, though further research is needed to assess its broader applicability.

**Background:**

Endotracheal intubation in patients with compromised airways is a notoriously complex and daunting task for anesthesiologists. Throughout the years, numerous supportive techniques and innovative equipment have been developed to address this challenge. This randomized clinical study sheds light on the potential benefits of utilizing an external pre-cricoid emitting infrared light source, the ‘Infrared Red Retrograde Intubation System’ (IRRIS^®^), which produces a flashing light that can be detected within the airway. By leveraging this technology, anesthesiologists may be able to identify the airway quicker and more accurately, both in terms of time and anatomical level, compared to relying solely on a video laryngoscope/ flexible bronchoscope.

## Introduction

 Endotracheal intubation remains the gold standard for airway management [1], but the procedure may be associated with potential challenges, especially in remote areas, with inexperienced staff, and in critically ill patients [2]. Esophageal intubation is a critical complication of emergency airway management in critically ill patients that occurs in 8% of all attempts [3]. The consequences of multiple intubation attempts can be severe and include bleeding, tissue swelling, and airway contamination, leading to significant morbidity and mortality [4–7]. Therefore, successful first-attempt intubation is crucial, and visualization of the larynx and glottic opening is key to achieving this goal [8–10].

Video laryngoscopy is a valuable tool that improves laryngeal visualization and increases first-pass success rates [1]. However, its limitations in certain circumstances, such as the presence of blood, secretions, vomitus, or fogging of the lens, can hinder its effectiveness [11, 12]. It is also operator-dependent and is limited by the handler’s experience [13, 14]. Moreover, laryngoscopy in the presence of highly contagious respiratory illnesses such as COVID-19 introduces additional challenges, including the need for personal protective equipment (PPE) and powered air-purifying respirators (PAPR), making tracheal intubation particularly challenging [15, 16].

Several adjuncts and maneuvers may help with difficult intubation, including, but not limited to, using a flexible, rigid, or malleable stylet within the tracheal tube, using transillumination systems such as Trachlight™ (Saturn Biomedical System Burnaby, BC, Canada) or the Surch-Lite™ (Bovie Medical Corporation, Clearwater, FL, USA) [17]. One significant limitation of the latter methods is the requirement of a specific laryngoscope/stylet during intubation to view the emitted light from within the lumen externally on the skin [18]. Practitioners experienced with other devices may find those particular adjuncts unfamiliar.

**Fig. 1:**
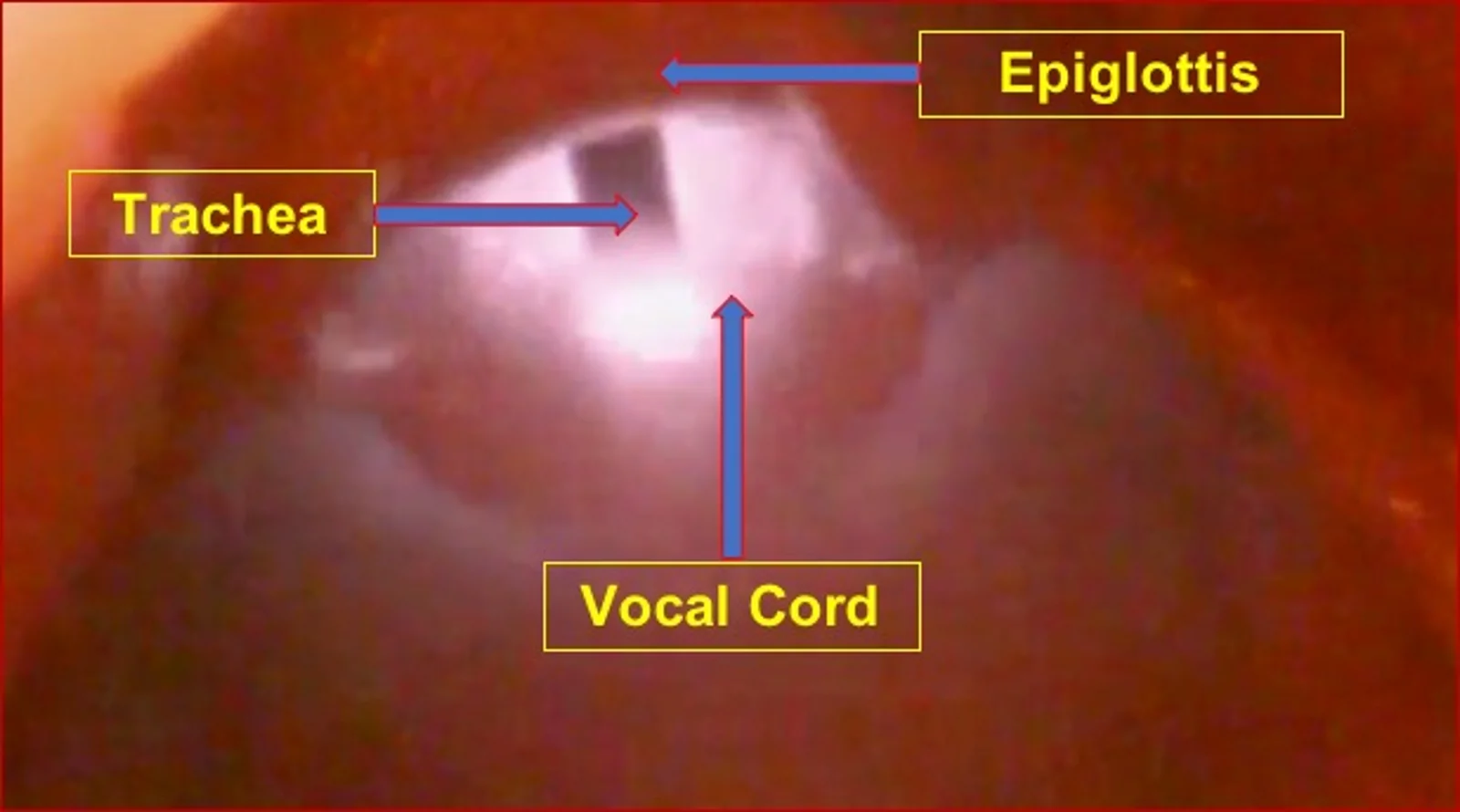
Image captured from the videolaryngoscope screen depicting infrared light from the IRRIS device illuminating the airway and providing clear visualization of the vocal cords and trachea

One alternative approach is the use of an infrared light device, which can penetrate tissues and provide a clear visual cue to guide intubation [19, 20]. The device is applied on the skin over the larynx and can be seen from within the tracheal lumen (hence the name ‘retrograde’ illumination) [20] **(**Fig. 1**).** By emitting infrared/near-infrared light (with a wavelength between 730 nm and 1000 nm) into the neck tissues, the ‘Infrared Red Retrograde Intubation System’ (IRRIS^®^) or Infrared Red Device (IRD) (Guide in Medical, Nazareth) creates a beacon-like effect within the airway [21]. The waxing and waning intensity of the light is detected by a video-endoscopic camera **(**Fig. 1**)**. This allows for clear visualization of the blinking light inside the trachea and glottis, even without infrared filters in the scope, enabling clinicians to distinguish the glottis from its surrounding structures with greater ease [18]. Since the emitted light is invisible, IRRIS is incompatible with direct laryngoscopy.

Although invisible to the naked eye, video systems convert infrared light into visible light, displaying it on their monitor screens. This feature enables the airway to be distinguished from the esophagus and adjacent tissue structures, preventing ‘false passage’ during tracheal tube advancement. After successful intubation, the IRRIS device is removed from the neck and discarded. This study aims to assess the feasibility and impact of IRRIS on video laryngoscopy performance, intubation time, and first-attempt success rate in comparison to video laryngoscopy alone, with the goal of improving airway management outcomes.

## Methodology

This is a single-center prospective randomized controlled pilot study conducted at the Ambulatory Care Center, Hamad Medical Corporation, Doha, Qatar. The primary outcome is the feasibility of IRRIS in the performance of video laryngoscopic tracheal intubation and its impact on first-pass success. Secondary outcomes are listed below:

1. Visibility of the glottic entrance.
2. Time to recognize the illuminated laryngeal inlet.
3. Number of intubation attempts.
4. Type of video laryngoscopy used and compatibility with IRRIS.
5. Alternative techniques of intubation.
6. Presence/severity of skin lesions attributed to the IRRIS device (discomfort, pressure injury, irritation, redness, burn).

The study was approved by the Medical Research Center, Hamad Medical Corporation, Doha, Qatar (MRC-01-21-185) and conducted with patients’ informed consent. The trial was registered prior to patient enrolment at clinicaltrials.gov (NCT04991545), and patients’ eligibility for the study was determined during preoperative visit.

The inclusion and exclusion criteria were the following:

Inclusion criteria:

- Adults above 18 years of age.
- Any general anesthesia requiring endotracheal intubation.
- All Mallampati scores 1–3.
- ASA physical status 1–3.

Exclusion criteria:

- Age below 18 years.
- Refusal or inability to sign the consent.
- Emergency cases.
- Rapid sequence induction.
- Maxillofacial abnormality or trauma.
- Impaired head and neck mobility.
- Scars or skin injuries at the neck.
- Pregnancy.
- Skin disorders and skin light sensitivity.

A total of 30 patients were enrolled in the study, a sample size deemed sufficient for our pilot cohort to determine whether further research is warranted. Group A (*n* = 15) was the control group, to whom intubation was performed without the IRRIS device. Group B (*n* = 15) comprised the intervention group, where the IRRIS device was used to aid video laryngoscopy.

**Fig. 2:**
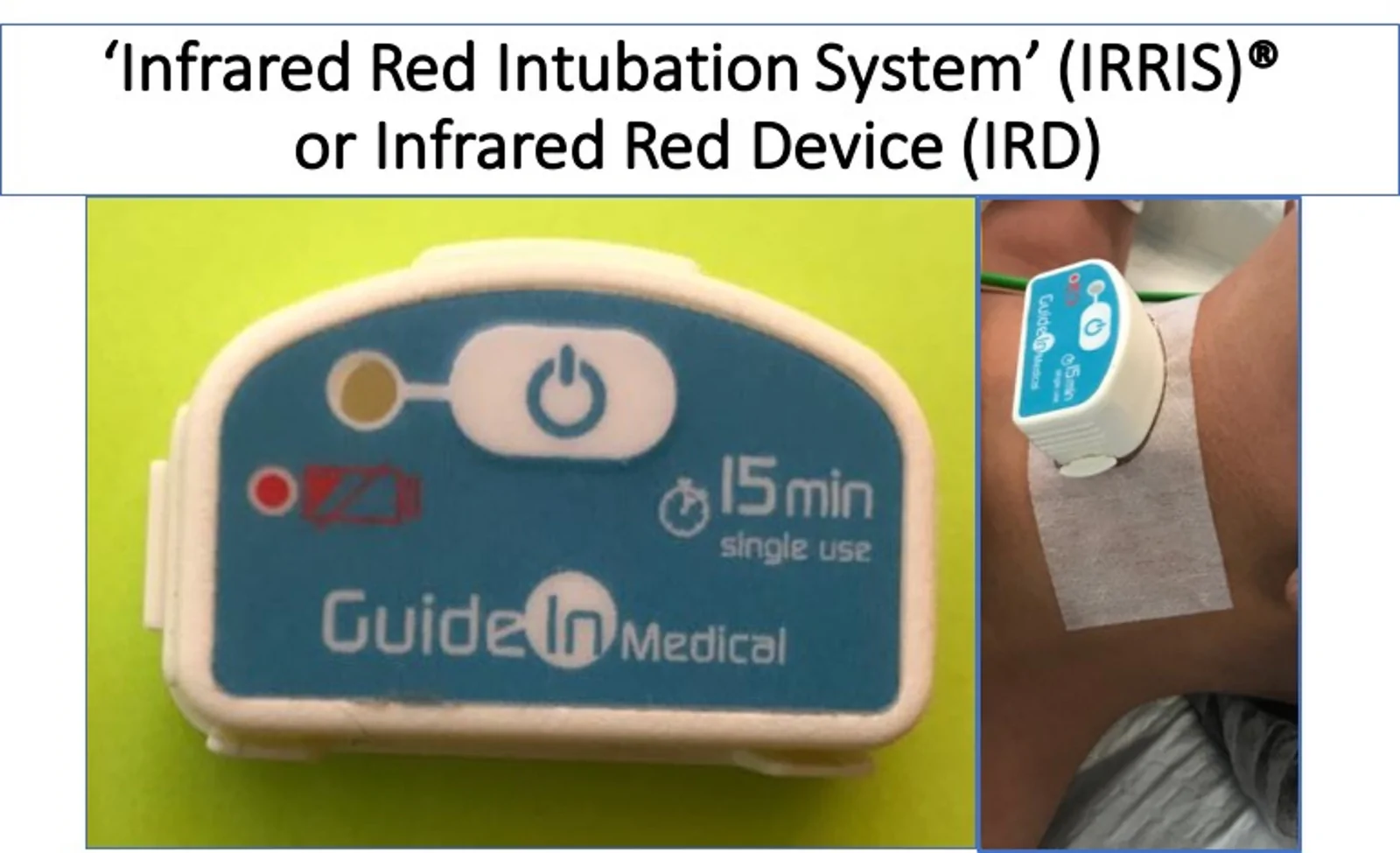
Picture of the IRRIS device with the turn on switch in the image on the left, and application of device to the anterior skin of the neck above the sternal notch in the image on the right

Randomization was implemented using sealed, opaque envelopes to ensure allocation concealment. The first operator, an experienced anesthetist skilled in video laryngoscopy, performed the endotracheal intubation. The second operator was responsible for opening the randomization envelope corresponding to the participant’s sequence and applying the IRRIS device to the anterior skin of the neck above the sternal notch **(**Fig. 2**)**. The IRRIS device was applied to all patients, however it was only activated in the intervention group (Group B), without informing the first operator to maintain blinding. This approach ensured that the allocation remained concealed until the point of randomization, minimizing selection bias and preserving the integrity of the trial. The IRRIS^®^ device is compact, disposable, and CE-marked (compliant with European Union (EU) safety, health, and environmental protection standards) corresponding to the size of a matchbox. It gets securely attached to the anterior neck skin, between the sternal notch and the thyroid cartilage, ideally over the cricothyroid membrane. Adhesive stripes on both sides of the device ensure a firm fixation on the skin [18].

After IRRIS application and ensuring the patient’s comfort, anesthesia was induced with fentanyl (1–2 micrograms/kg), propofol (2 mg/kg), or ketamine (1–2 mg/kg), and rocuronium (0.8–1.2 mg/kg). Once complete relaxation was achieved, as confirmed by a train-of-four (TOF) of 0 using a nerve stimulator, the first operator performed laryngoscopy using a video laryngoscope of their choice (Glidescope [Verathon Medical, BC, Canada], C-Mac [Karl Storz, Germany] or HugeMed [Shenzhen, Guangdong, China]) to insert the tracheal tube. All endotracheal tubes were standardized, with size 7.0 mm used for female patients and size 7.5 mm for male patients. The tube was armed with a malleable stylet and molded to a curved ‘hockey-stick’ shape or the same curve as the laryngoscope blade. Following successful tracheal intubation, the IRRIS device was carefully removed and discarded. To assess potential skin irritation, postoperative checks of the neck skin condition were conducted at three distinct time intervals after IRRIS device removal: immediately after removal, 2 h post-removal, and 24 h post-removal. All interventions adhered to the Difficult Airway Management guidelines (CG10166) of our local hospital and aligned with the Difficult Airway Society guidelines.

The intubating person (first operator) was responsible for observing and collecting the following parameters:

1. Was intubation successful on the first attempt? (Yes or No)
2. Can the illuminated laryngeal structure be easily differentiated from adjacent structures, such as the esophagus? (Yes or No)
3. What is the Percentage of Glottic Opening (POGO) score of the video-laryngoscopic view, ranging from 1% to 100%?
4. How visible is the glottic entrance, rated on a subjective visual analogue scale (VAS) from 0 (extremely poor visibility) to 10 (excellent visibility)?
5. What is the time taken to identify the illuminated laryngeal inlet after inserting the video laryngoscope (VL), measured in seconds?
6. How credible is the IRRIS in identifying the correct intubation pathway, rated on a scale from 1 (not credible at all) to 10 (very credible)?
7. Were there any secretions, blood, or vomitus present in the upper airway?

The second operator was responsible for removing the IRRIS device from the neck and discarding it safely, as well as monitoring intubation performance and documenting the following parameters:

1. Type of video laryngoscopy used.
2. What type of video laryngoscope blade was used: channeled or non-channeled and angulated or non-angulated?
3. Was tracheal intubation via video-laryngoscopy accomplished within the 20-second time frame? (Yes or No)
4. What is the duration from VL insertion to tracheal tube cuff inflation, measured in seconds?
5. How difficult was intubation, rated on a subjective scale from 1 (extremely easy) to 10 (extremely challenging)?
6. Was additional external assistance, such as pressure or manipulation of the head or neck, necessary to aid video laryngoscope insertion and intubation?
7. If initial intubation attempts (if more than one attempt) with IRRIS failure, what was the alternative airway securing method/technique?

Demographic data, vital parameters, and clinical data were collected from electronic patient records maintained using the Cerner Electronic Patient Records System (Citrix Systems, Fort Lauderdale, Florida, USA). To ensure confidentiality, study IDs were used in place of identifiable information. Data was entered into a secure, password-protected database with restricted access, available only to the research team. Each participant was assigned a unique alphanumeric study ID. At the end of the study, the link between the identifiers and study codes was deleted, and the anonymized dataset was retained for a minimum of five years in accordance with local policy.

The study’s categorical data were summarized as frequencies and percentages, while interval data were presented as medians and interquartile ranges (IQRs). Normally distributed data were analyzed using two-tailed unpaired Student’s t-tests. For continuous variables with skewed distributions, Mann-Whitney U tests were employed, and dichotomous variables were analyzed using Fisher’s exact tests. A p-value of less than 0.05 was considered statistically significant.

All data analyses were performed using SPSS version 26 (IBM Corp, Armonk, NY, USA), and tables were created using GraphPad Prism version 5.0a (GraphPad Software, San Diego, CA, USA). On occasion, quick statistical calculations for select datasets were performed using Llama by Meta (1 Hacker Way, Menlo Park, CA, USA), accessed through Perplexity Labs (San Francisco, CA, USA).

## Results

Baseline characteristics are shown in Table 1. There was great homogeneity between the two groups in terms of physical status and airway examination. All tracheal intubations were successfully accomplished with the initial video laryngoscopy blade of choice in both groups, and no alternative techniques were required. The use of IRRIS was successful and without side effects in all patients in the treatment group. No patient described any discomfort when awake. Only one patient complained of severe sore throat two hours and 24 h postoperatively (Table 2), while the majority of patients reported mild to moderate soreness.

**Table 1:** Baseline characteristics: a number, a median (IQR [Range]), or mean (SD)

Parameter	Group	
	A	B
Sex; M/F	9/6	10/5
Age; years	33 (14 [23–68])	41 (12 [19–62])
Body mass index (BMI); kg.m^−2^	29.1 (4.5 [20.8–51.82]	29.5 (5.1 [16.4–37])
Neck Mobility (Good/Restricted)	15/0	14/1
Mouth Opening; cm	4.067 (0.5627)	3.9 (0.8062)
Thyromandibular (TM) distance; cm	6.167 (0.3086)	6.3 (0.7973)
Mallampati grade distribution; 1/2/3/4	4/7/5/0	4/11/1/0
ASA status; 1/2/3	5/9/1	7/7/1

**Table 2.** Sore throat 2 h and 24 h postoperatively

2 h postoperatively				
Severity	NIL	Mild	Moderate	Severe
Group A	8	1	6	0
Group B	12	0	2	1

24 h postoperatively				
Severity	NIL	Moderate	Severe	
Group A	10	5	0	
Group B	9	5	1	

Surgical procedures are listed in Table 3. The study center offers general and otorhinolaryngology surgeries in an ambulatory (day-case) setting. The otorhinolaryngology pool was selected because it presented a higher likelihood of varying degrees of airway difficulty compared to other specialties, which added significant value to the study. The case mix represented a wide range of procedures, from simple biopsies to more complex surgeries such as micro-laryngoscopy. This variety in surgical cases also necessitated the use of different types of endotracheal tubes (micro-laryngeal, armored, and standard), exposing the IRRIS technique to more variable and real-world scenarios.

**Table 3:** Surgical procedures

	Hemi-thyroidectomy	Microlaryngoscopy	Nasal septoplasty	Nasopharyngeal biopsy	Radical mastoidectomy	Septoplasty	Submandibular gland excision	Tongue biopsy	Tonsillectomy	Tympanoplasty
Group A	1	4	0	1	1	1	1	1	4	1
Group B	0	9	1	0	0	2	0	0	2	1
Total	1	13	1	1	1	3	1	1	6	2

Surgeons’ satisfaction was measured and shown in (Table 4). The main parameter was the time consumed to get the patient ready for the World Health Organization (WHO) Surgical Safety Checklist and, therefore, starting the surgery. This metric was particularly significant, as delays in preparation could adversely affect the overall performance of the surgical team.

**Table 4:** Surgeons’ satisfaction

Grade	Medium	Good	Very Good	Excellent
Group A	4	7	4	0
Group B	0	4	6	5

Intubation performance demonstrated nearly identical percentages of glottic opening (POGO) and glottic entrance visibility between the two groups. However, intubation time—measured from the insertion of the scope into the patient’s mouth to cuff inflation—was significantly longer in the IRRIS group, with an average difference of 10 s. Additionally, more cases in the IRRIS group required external manipulation to adjust positioning. In the control group, the blades used represented a balanced mix of the three video laryngoscopes, whereas in the IRRIS group, the C-Mac D-Blade was predominantly utilized. In the intervention group, the credibility and helpfulness of the IRRIS were rated as average, with a wide range of variability among patients. The extremes in ratings, both high and low, were not associated with specific patient characteristics.

Retrograde transillumination by IRRIS effectively highlighted the laryngeal inlet on the screen in all patients in the treatment group, while other structures—particularly the esophagus—remained significantly darker. In the most obese patient in our cohort (BMI of 46 kg/m² and neck circumference of 56 cm), the vocal cords were not visible without IRRIS due to abundant supraglottic tissue, which left the glottis unrecognizable. In this specific case, IRRIS clearly indicated the correct pathway.

Tracheal intubation was successfully performed in all patients using the initially intended technique. There were notable differences in both objectively measured parameters, such as intubation duration and subjective assessments of difficulty level. No patients reported discomfort during the awake period prior to anesthesia induction, while the IRRIS device was positioned on the anterior neck surface.

## Discussion

Anterograde light-guided intubation using an illuminated stylet has been utilized in the past with varying degrees of success [21–25]. However, only one research group has reported the use of a retrograde transillumination technique, though it employed visible light, which does not specifically highlight the airway [26].

Commercial anterograde transillumination devices, such as the Surch-Lite™ (Bovie Medical Corporation, Clearwater, FL, USA) and the previously manufactured Trachlight™ (Saturn Biomedical System, Burnaby, BC, Canada) stylets, can be considered predecessors to the IRRIS. However, these devices have notable limitations: they require appropriately dimmed ambient lighting conditions, which may not always be feasible, and their effectiveness is significantly influenced by the thickness of the anterior neck tissues [18, 24, 25].

Attempting to transmit visible light from the anterior neck surface to the larynx for visualization during direct laryngoscopy might initially appear promising. However, this approach is technically unfeasible because visible wavelengths cannot adequately penetrate or selectively illuminate the airway structures [20]. In contrast, infrared and near-infrared light demonstrate superior penetration into hollow organs such as the trachea, explicitly highlighting the glottis when used with a video endoscope [20]. In order to achieve this, the endoscope must convert the “invisible” infrared light to visible light on the device’s video screen. Consequently, the IRRIS device is designed to function exclusively with video laryngoscopes as well as flexible and rigid video endoscopes that do not have built-in infrared filters [18]. According to the manufacturer, many other video-endoscopic devices can be modified to display the infrared light emitted by IRRIS on their screens. However, it is essential for the user to ensure that the chosen video-endoscopic device is compatible with infrared visualization before utilizing the IRRIS technique [18].

Our pilot study aimed to evaluate the feasibility and impact of the IRRIS on video laryngoscopy performance, intubation time, and first-attempt success rate compared to video laryngoscopy alone. The results indicated that all tracheal intubations in both groups were successful, encompassing a wide range of BMIs and Mallampati grades (1–3). The majority of surgeons reported good to very good satisfaction, with five surgeons expressing excellent satisfaction in the IRRIS group, suggesting positive cooperation among surgical team members. Additionally, the IRRIS^®^ device demonstrated safety, as no significant adverse reactions were observed in patients up to 24 h postoperatively.

Despite similar intubation performance and glottic entrance visibility between the two groups, the subjective level of difficulty during intubation was slightly higher in Group B compared to Group A (Table 5), and intubation time was significantly longer in the IRRIS group, with an average difference of 10 s. This difference may be attributed to the higher presence of airway secretions in Group B, as well as the unfamiliarity with the IRRIS technique and the learning curve required to master the new inputs involved in the intubation process.

**Table 5:** Intubation performance number or median (IQR [range])

Parameter	Group	
	A	B
First-attempt intubation success? (Y/N)	15/0	15/0
Easy laryngeal structure differentiation? (Y/N)	N/A	15/0
POGO score (%)	80% (0% [60% − 100%])	80% (10% [60% − 100%])
Glottic entrance visibility (VAS 0–10)	7 (2 [4–10])	7 (3 [4–10])
Time to identify illuminated laryngeal inlet (seconds)	N/A	21 (3 [18–25])
IRD helpfulness in detection of airway structures (VAS 0–10)	N/A	6 (2 [4–9])
IRD credibility (1–10)	N/A	5 (2 [2–8])
Intubation difficulty (VAS 0–10)	5 (2 [3–9])	8 (2 [5–10])
Upper airway secretions/blood/vomit present? (Y/N)	1/14	3/12
Intubation time (time to cuff inflation in seconds)	18 (4 [15–29])	28 (5 [20–36])
External assistance (No/External Pressure/Head Manipulation)	(10/4/2)	(3/4/7)
Alternative airway method (if IRRIS fails)	N/A	0
Type of video laryngoscopy (HugeMed/Glidescope/C-Mac [C-Blade/D-Blade])	(2/5/9[3/6])	(1/0/14[3/11])

It is important to note that these performance metrics were recorded from an experienced user. This “best user” research design was employed to eliminate personal competence as a variable in the clinical evaluation of IRRIS. However, individual ability to recognize anatomy remains an important factor, and it will be crucial to assess whether novices find the procedure beneficial and easy to adopt.

Our pilot study demonstrates the clinical feasibility of IRRIS in elective surgical cases, as evidenced by successful recruitment and randomization, straightforward device application, absence of adverse events, and integration into standard workflow without disruption —fulfilling key feasibility domains highlighted in the Consolidated Standards of Reporting Trials (CONSORT) extension for pilot and feasibility trials [27]. These positive feasibility outcomes are clinically significant, as they provide the foundation for a follow-up feasibility trial specifically evaluating IRRIS in patients with anticipated or established difficult airways. A trial as such would assess acceptability, safety, and practical benefit in high-risk scenarios, and would support the design of a multicenter randomized controlled trial (RCT) to determine the clinical effectiveness of IRRIS in improving first-pass success and reducing airway-related morbidity.

## Limitations

This is a pilot study on a limited number of patients, which might not represent all the possible airway conditions in a given population. It also specifically excluded any patient with airway abnormalities to assess the device in ‘optimum’ conditions. Further studies on the usability and benefits of IRRIS in patients with difficult airway conditions might be of value.

## Conclusion

The usability and added value of IRRIS for intubation have been well-documented in the literature [18, 20, 28]. However, the approach in our study was to compare the IRRIS system with standard video laryngoscopy and determine whether the first-pass success rate of video laryngoscopes could be enhanced and optimized. Our findings demonstrated that IRRIS is effective for retrograde transillumination of the larynx when viewed with a compatible video laryngoscope, clearly highlighting the intubation pathway in comparison to nearby structures such as the esophagus. The IRRIS enhances the differentiation of the intubation route by selectively illuminating relevant structures without causing discomfort or harm to patients undergoing video laryngoscopic intubation facilitated by this innovative retrograde transillumination device. Our findings establish feasibility and safety in elective patients and justify a dedicated feasibility trial in difficult airway cases, in line with CONSORT guidance, to support the design of a larger multicenter RCT.

## Data Availability

No datasets were generated or analysed during the current study.
